# Different Rhizospheric pH Conditions Affect Nutrient Accumulations in Rice under Salinity Stress

**DOI:** 10.3390/plants10071295

**Published:** 2021-06-25

**Authors:** Mami Nampei, Kamonthip Jiadkong, Sumana Chuamnakthong, Thanakorn Wangsawang, Tanee Sreewongchai, Akihiro Ueda

**Affiliations:** 1 Graduate School of Integrated Sciences for Life, Hiroshima University, 1-4-4 Kagamiyama, Higashi-Hiroshima, Hiroshima 739-8528, Japan; d213700@hiroshima-u.ac.jp (M.N.); d210776@hiroshima-u.ac.jp (K.J.); 2 Graduate School of Biosphere Science, Hiroshima University, 1-4-4 Kagamiyama, Higashi-Hiroshima, Hiroshima 739-8528, Japan; sue_mana@hotmail.com; 3 Faculty of Agricultural Technology, Valaya Alongkorn Rajabhat University under the Royal Patronage, 1 Moo 20, Phaholyothin Road, Klong Neung, Klong Luang, Pathum Thani 13180, Thailand; pug_thanakorn03@hotmail.com; 4 Department of Agronomy, Faculty of Agriculture, Kasetsart University, Bangkok 10900, Thailand; agrtns@ku.ac.th

**Keywords:** Na exclusion, nutrients accumulation, rice saline-alkaline tolerance

## Abstract

This study was conducted to determine the responses to saline-alkaline (SA) stress with regard to nutrient accumulation in two rice varieties having different tolerances to salt-stress. A salinity-tolerant landrace, Pokkali, and a salinity-sensitive variety, PTT1, were exposed to three levels of SA conditions, pH 7.0 (mild), pH 8.0 (moderate), and pH 9.0 (severe), under 50 mM Na stress. The results indicated that Pokkali had comparably greater SA tolerance than PTT1 owing to its higher biomass production. The maintenance of the lower Na/K ratio in Pokkali shoots was achieved by the higher expression of *OsHKT1;5* encoding a Na^+^ transporter in the shoots, *OsNHX1* encoding a tonoplast-localized Na^+^/H^+^ antiporter in the roots, and *OsHAK16* encoding a K^+^ transporter in the roots under SA conditions. We propose that the high expression of Fe deficiency-responsive genes, *OsIRT1*, *OsIRO2*, *OsYSL15*, *OsNAS1*, and *OsNAS2*, in both rice varieties under all SA conditions should contribute to Fe homeostasis in the shoots. In addition, SA treatment increased the concentrations of Ca, Mn, Zn, and Cu in the roots but decreased their concentrations in the shoots of both varieties. Overall, the results indicated that high rhizospheric pH influenced nutrient uptake and translocation from the roots to the shoots in rice.

## 1. Introduction

Salt-affected soils, including those affected by a large quantity of Na, are harmful to agricultural crops, such as rice. The presence of these minerals leads to the classification of soil as either saline or sodic. In arid and semi-arid areas, soil sodicity is accompanied by soil salinization, in which case the soil becomes a saline-sodic soil, which has poor characteristics typical of both salt-affected soils. Sodic and saline-sodic soils contain alkaline salts, such as NaHCO_3_ and Na_2_CO_3_, and their soil pH is above 8.5. The sodic soils are estimated to make up 617.3 million ha globally, which is equivalent to 4.1% of the land area [1].

Salt-stress is a complicated stress consisting of osmotic stress, the first phase, and ionic stress, the second phase [2]. Osmotic stress is caused by increasing Na concentrations in the rhizosphere, which in turn decreases the water potential and prevents plants from absorbing water through their roots [2,3]. Ionic stress occurs due to excess Na flowing into the plant body. When Na flows into the plant body, the plants activate salinity-tolerance mechanisms to avoid Na damage. A Na^+^/H^+^ antiporter, SOS1, which is located on the plasma membrane of the roots, eliminates Na flowing into the roots, whose activity is increased under high Na conditions [4,5]. Moreover, in the roots, HKT1, a high-affinity K^+^ family, plays a significant role in excluding Na from the xylem vessel [2]. In rice, OsHKT1;5 exists in parenchyma cells adjacent to the xylem vessel and the expression of *OsHKT1;5* is upregulated under salt-stress conditions [6,7]. Na flowing from the roots to the shoots is excluded from the xylem by OsHKT1;4 and OsHKT1;5 in the leaf sheath [7,8,9,10]. These sequential salinity-tolerance mechanisms prevent Na from flowing into the leaf blade where photosynthesis and metabolism occur. In addition, to protect cytoplasmic enzymes from Na damage, Na in the cytoplasm of the roots and shoots is sequestered in vacuoles through the action of a K^+^/Na^+^ transporter of the NHX family located on the tonoplast to maintain K/Na homeostasis in the cytoplasm [11,12,13,14]. Aside from the regulation of Na transport, several genetic loci controlling salinity-tolerance in rice have also been identified such as *saltol QTL* [15,16].

Salt-stress inhibits nutrient absorption in plants. K, which is the most abundant inorganic element in plants, is an alkali metal element in the same family as Na. The absorption of K competes with that of Na in the roots under salt-stress conditions [5,17,18]. Since excess Na inhibits the physiological activity of K, maintaining a lower Na/K ratio is important for preserving plant life [2,19]. Plants absorb K through the action of the AKT1 and HAK families. The expression of *OsHAK5* and *OsHAK16* is also promoted under low K conditions in rice. Furthermore, these genes are upregulated well under salt-stress conditions [18,20,21]. On the other hand, although the expression of *OsHAK1* is stimulated under low K conditions, it is downregulated under low K and high Na conditions, thus suggesting that *OsHAK1* does not contribute to the maintenance of K homeostasis under salt-stress conditions [21].

Since Fe is an essential element for chlorophyll synthesis and is important for maintaining photosynthesis in plants [22], plants exposed to low Fe conditions activate Fe acquisition and absorption mechanisms. In addition, most gramineous plants have a strategy II system for Fe acquisition and absorption, whereas rice has strategy II and partial strategy I systems [23,24,25]. The strategy II system is utilized to acquire Fe^3+^ by secreting mugineic acids (MAs) in the rhizosphere [25,26]. This system has been observed in gramineous plants [23]. Plants secrete MAs from the roots, which form complexes with Fe^3+^ in the rhizosphere. The Fe(III)-MAs complexes formed are then absorbed via Fe transporters on the plasma membrane in the roots [27]. In rice, under low Fe conditions, the *OsYSL15* expression is upregulated, leading to the acquisition of Fe(III)-DMA (deoxymugienic acid) complexes [28]. In *Arabidopsis thaliana*, Fe^2+^ is acquired via Fe^2+^ transporters, IRT1, and IRT2, after reducing Fe^3+^ to Fe^2+^, whereas in rice only IRT1 plays this primary role, and Fe^2+^ is directly absorbed via IRT1 from the soil [24,29,30].

In the previous studies, the mechanisms of salinity tolerance have been identified under neutral salt-stress conditions; however, only a few studies have been conducted on the mechanisms of tolerance to alkaline salt-stress [31,32]. The research conducted by Chunamnakthong et al. indicated that rice accumulated more Na at pH 8.0 than at pH 7.0 under SA conditions, suggesting that high-pH promotes Na accumulation in rice [31]. Meanwhile, Li et al. reported that the Fe concentrations in rice shoots were reduced after exposure to SA stress and that SA tolerant rice Fe acquisition was not influenced so much by SA stress [32]. However, several questions on the SA tolerance mechanisms of rice are yet to be addressed, including the effects of SA stress on essential element absorption in rice. To successfully cultivate rice on a SA-damaged land, it is important to understand the mechanisms underlying SA tolerance and breed rice cultivars having improved SA tolerance without growth penalties. Therefore, this study determined how SA stress affects essential element absorption using a salinity-tolerant variety, Pokkali, and a salinity-sensitive variety, PTT1.

## 2. Results

### 2.1. Plant Growth

**Figure 1:**
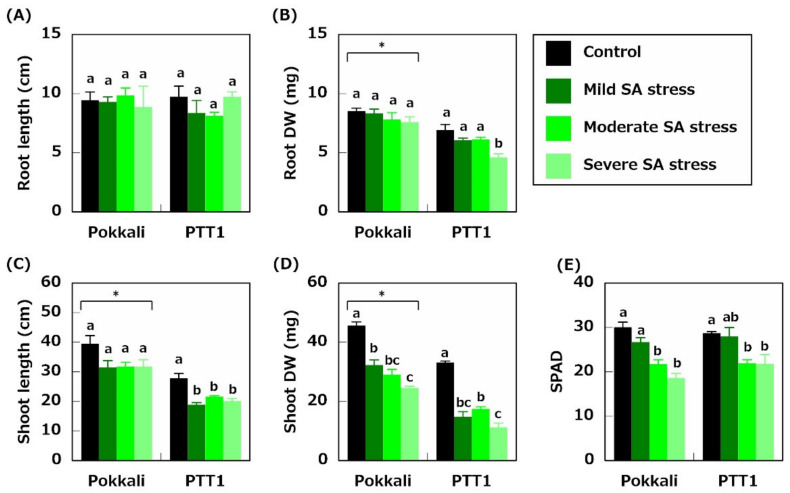
Effects of saline-alkaline stress (mild: 50 mM Na + pH 7.0, moderate: 50 mM Na + pH 8.0, and severe: 50 mM Na + pH 9.0) on root length (**A**), root dry weight (DW) (**B**), shoot length (**C**), shoot DW (**D**), and SPAD value (**E**) of Pokkali and PTT1. Data are the means of 4 replicates ± S.E. Similar letters indicate no significant differences between the treatments in each variety (*p* < 0.05). Asterisks show significant differences between Pokkali and PTT1 in each treatment (*p* < 0.05).

To identify the effects of SA stress on the two rice varieties, we measured specific physiological parameters. Twelve-day-old Pokkali (salinity-tolerant rice variety) and PTT1 (salinity-sensitive rice variety) were treated under mild SA (50 mM Na + pH 7.0), moderate SA (50 mM Na + pH 8.0), or severe SA (50 mM Na + pH 9.0) stress conditions. The root length was significantly unaffected by SA stress (Figure 1A). The dry weight (DW) of Pokkali roots did not also differ between treatments, whereas the DW of PTT1 roots decreased by 33.4% under severe SA stress conditions (Figure 1B). The difference in the shoot length of Pokkali was also insignificant between the controls and SA stress conditions (Figure 1C). However, that of PTT1 was reduced under SA stress conditions (Figure 1C). Furthermore, the DW of Pokkali shoots gradually decreased as pH increased (45.6, 32.3, 29.1, and 24.6 mg in the control, mild, moderate, and severe SA stress conditions, respectively) (Figure 1D). The shoot DW was lower under mild SA stress treatments in PTT1 (14.7 mg) than the control (33.7 mg) (Figure 1D). The shoot DW of PTT1 under moderate and severe SA stress conditions, however, was similar to that under mild SA stress conditions (Figure 1D). In addition, the SPAD value of Pokkali and PTT1 decreased under moderate (27.7% and 23.7%) and severe (38.0% and 24.0%) SA stress conditions, but not under mild SA stress conditions (Figure 1E).

### 2.2. Ion Concentration

**Figure 2:**
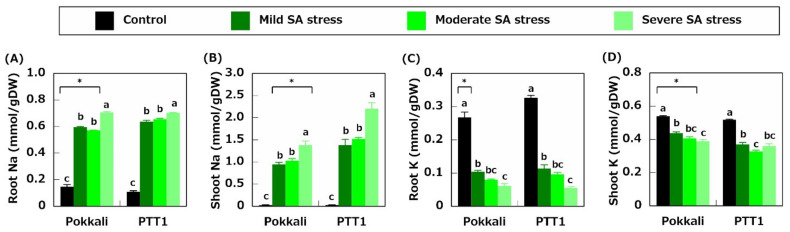
Effects of saline-alkaline stress (mild, moderate, and severe) on the Na concentration in the roots (**A**) and shoots (**B**) and the K concentration in the roots (**C**) and shoots (**D**) of Pokkali and PTT1. Data are the means of 4 replicates ± S.E. Similar letters indicate no significant differences between the treatments in each variety (*p* < 0.05). Asterisks show significant differences between Pokkali and PTT1 in each treatment (*p* < 0.05).

To reveal the effects of SA stress on nutrient absorption, the ion concentrations in the salinity-tolerant Pokkali and salinity-sensitive PTT1 varieties were measured. The Na concentrations in Pokkali and PTT1 roots increased 4- and 5.8-fold under mild SA stress conditions, respectively; therefore, it was confirmed that the Na concentrations increased with the increase in pH (Figure 2A). Furthermore, the Na concentrations in Pokkali roots were lower than those in PTT1 roots under mild and moderate SA stress conditions, which were comparable to those of PTT1 under severe SA stress conditions (Figure 2A). Compared with the control conditions, the Na concentrations in the shoots of Pokkali and PTT1 increased by 35.2- and 47.5-fold under mild SA stress conditions, respectively (Figure 2B). Under moderate and severe SA stress conditions, the Na concentrations in Pokkali shoots increased by 38.2- and 51.3-fold, whereas increased by 52.5- and 75.9-fold in PTT1, respectively (Figure 2B). Moreover, the Na concentrations of PTT1 shoots subjected to SA treatments were significantly higher than those of Pokkali (Figure 2B).

The K concentrations in Pokkali and PTT1 roots were significantly reduced under mild SA stress conditions (61.2% and 64.2%, respectively) and decreased with the increase in pH (Figure 2C). Under severe SA stress conditions, both rice varieties had lower K concentrations in the roots by 77.1% (Pokkali) and 83.1% (PTT1), respectively, than the control (Figure 2C). The shoot K concentrations tended to decrease as SA stress worsened (Figure 2D). Meanwhile, under all conditions, the K concentrations of Pokkali shoots were higher than those of PTT1 (Figure 2D). In addition, the Na/K ratio in the roots and shoots of both rice varieties increased under SA stress conditions, especially under severe SA stress conditions (Table 1). However, in the shoots, the Na/K ratio of PTT1 was higher than that of Pokkali under each SA stress conditions (Table 1).

**Figure 3:**
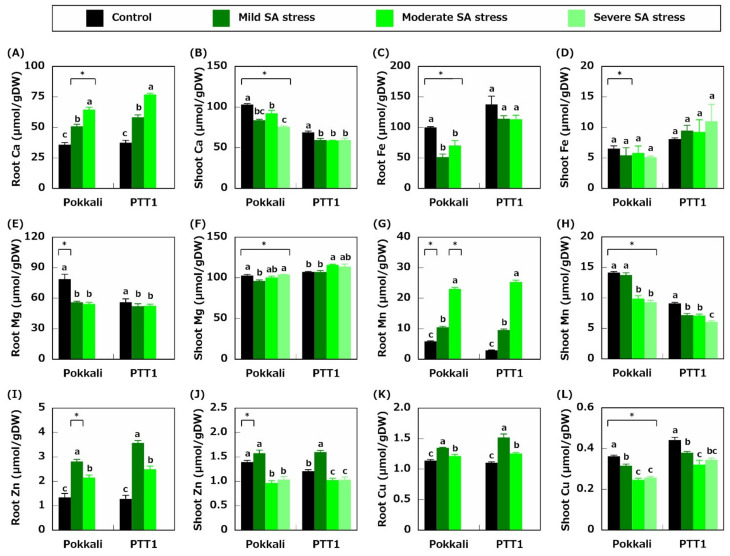
Effects of saline-alkaline stress (mild, moderate, and severe) on the Ca concentration of the roots (**A**) and shoots (**B**), Fe concentration of the roots (**C**) and shoots (**D**), Mg concentration of the roots (**E**) and shoots (**F**), Mn concentration of the roots (**G**) and shoots (**H**), Zn concentration of the roots (**I**) and shoots (**J**), and Cu concentration of the roots (**K**) and shoots (**L**) of Pokkali and PTT1. The concentration of each element in the roots of both rice varieties under severe SA conditions was not determined because of divalent metals deposition on the root surface. Similar letters indicate no significant differences between the treatments in each variety (*p* < 0.05). Asterisks show significant differences between Pokkali and PTT1 in each treatment (*p* < 0.05).

We also measured the Ca, Fe, Mg, Mn, Zn, and Cu concentrations. The results showed that under severe stress conditions, the root Ca concentrations were higher under SA stress conditions in Pokkali and PTT1 than in the control (Figure 3A). In particular, the Ca concentrations of Pokkali and PTT1 roots under moderate SA stress conditions were 1.8- and 2.1-fold higher than those of the control, respectively (Figure 3A). Contrarily, the shoot Ca concentrations decreased under SA stress conditions in both rice varieties (Figure 3B). However, no statistical difference was observed in the shoot Ca concentrations between varieties across all SA treatments (Figure 3B). The root Fe concentrations also decreased under mild and moderate SA stress condition sin Pokkali (48.7% and 29.9%, respectively) compared with the control (Figure 3C). Although there was no statistically significant difference, a trend similar to those of Pokkali was observed in PTT1 roots (Figure 3C). However, no significant difference was observed in the shoot Fe concentrations in both rice varieties across treatments (Figure 3D). In Pokkali roots, the Mg concentrations were lower under mild (29.0%) and moderate (31.0%) SA stress conditions, whereas in PTT1 roots, the Mg concentrations under mild and moderate SA stress conditions slightly declined (7.0% and 6.5%, respectively) (Figure 3E). Additionally, the shoot Mg concentration decreased slightly under mild SA treatments in Pokkali (6.5%) compared with that in the control (Figure 3F). However, in PTT1 shoots, the Mg concentration increased slightly under moderate SA stress (1.1-fold) conditions (Figure 3F). The root Mn concentrations increased by 3.9-fold in Pokkali and 8.6-fold in PTT1 under moderate SA stress conditions (Figure 3G). Contrarily, the shoot Mn concentrations in both rice varieties decreased with the increase in SA severity (Figure 3H). Moreover, the root Zn concentrations in Pokkali and PTT1 under mild SA stress conditions were the highest (0.18 and 0.23 mg/g DW, respectively) between all treatments (Figure 3I). Though Pokkali was not statistically different, the shoot Zn concentrations in Pokkali and PTT1 under mild SA stress conditions were the highest between all treatments (1.1- and 1.3-fold higher than that in the control) (Figure 3J). Under moderate SA stress conditions, the root Zn concentrations in Pokkali and PTT1 were higher than those in the control (1.6- and 1.9-fold higher, respectively), whereas those in the shoots were lower (0.7- and 0.8-fold lower, respectively) (Figure 3I,J). There was no significant difference in the shoot Zn concentrations between the rice varieties under moderate and severe SA stress conditions (Figure 3J). Furthermore, the root Cu concentrations in Pokkali and PTT1 under mild SA stress conditions were the highest (1.2- and 1.4-fold, respectively) between all treatments, similar to the root Zn concentrations of both rice varieties (Figure 3K). SA stress also reduced the shoot Cu concentrations in Pokkali and PTT1. In particular, the shoot Cu concentrations in both varieties were lower under moderate SA stress conditions (32.0% and 27.3%, respectively) than the control conditions (Figure 3L).

### 2.3. Gene Expression Related to Na^+^ Transport

To compare the Na^+^ transport mechanisms in salinity-tolerant Pokkali and salinity-sensitive PTT1 under SA stress conditions, qRT–PCR analysis was conducted to examine the expression of genes related to the Na^+^ transport. The relative expression levels of each gene were calculated based on those of Pokkali under the control conditions.

**Figure 4:**
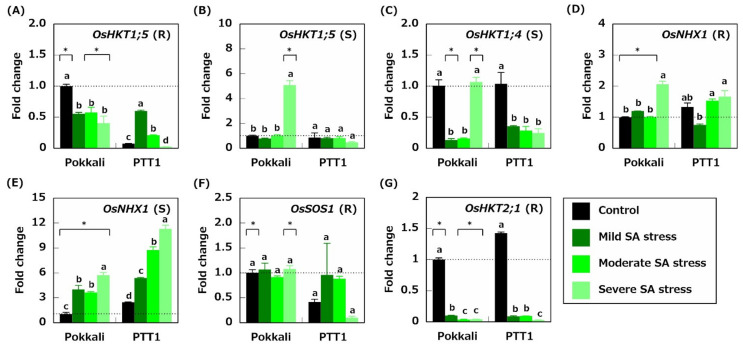
Relative expression of genes encoding Na^+^ transporters (*OsHKT1;5* and *OsHKT1;4*)*,* a K^+^/Na^+^ transporter (*OsNHX1*), and a Na^+^/H^+^ antiporter (*OsSOS1*). *OsHKT1;5* in the roots (**A**) and shoots (**B**), *OsHKT1;4* in the shoots (**C**), *OsNHX1* in the roots (**D**) and shoots (**E**), *OsSOS1* in the roots (**F**), and *OsHKT2;1* in the roots (**G**) of Pokkali and PTT1. The number of gene transcripts is evaluated based on that of Pokkali under control conditions. Similar letters indicate no significant differences between the treatments in each variety (*p* < 0.05). Asterisks show significant differences between Pokkali and PTT1 in each treatment (*p* < 0.05).

Na^+^ transporters, OsHKT1;4 and OsHKT1;5, restricted the accumulation of Na in the leaf sheaths and roots, respectively [7,8,9,10]. The results showed that the levels of the *OsHKT1;5* expression in PTT1 roots under the control conditions were only 0.1-fold those of the *OsHKT1;5* expression in Pokkali roots under the control conditions (Figure 4A). The *OsHKT1;5* expression was at similar levels between Pokkali and PTT1 roots under mild SA stress conditions, whereas under moderate and severe SA stress conditions, the expression in Pokkali roots was 2.4- and 16.4-fold higher than that in PTT1 roots, respectively (Figure 4A). These results indicate that Pokkali had higher Na exclusion activity in the shoots. The *OsHKT1;5* expression in Pokkali shoots under severe SA stress conditions was 5.1-fold higher than that under the control conditions. In contrast, the *OsHKT1;5* expression in PTT1 shoot under severe SA stress conditions was downregulated by 44.7% compared with PTT1 shoots in the control (Figure 4B). Furthermore, the *OsHKT1;4* expression in the shoots was inhibited under mild and moderate SA stress conditions in both rice varieties (Figure 4C). Under severe SA stress conditions, the *OsHKT1;4* expression in Pokkali was at the same level as the control conditions, whereas the expression in PTT1 was 72.1% lower than that under the control conditions (Figure 4C).

The functions of OsNHX1 are related to the Na^+^ compartmentalization into vacuoles and therefore contribute to the enhancement of tissue tolerance to excess Na accumulation [11,12,13,14]. Severe SA stress increased the *OsNHX1* expression in the roots of Pokkali by 2-fold compared with that in the roots of Pokkali under the control conditions (Figure 4D). Furthermore, while the *OsNHX1* expression in PTT1 roots under mild SA stress conditions was 56.6% that of PTT1 roots under the control conditions, the expression patterns of *OsNHX1* under moderate and severe SA stress conditions were similar to those of PTT1 under the control conditions (Figure 4D). In addition, the *OsNHX1* expression was significantly upregulated under SA stress conditions in the shoots of both rice varieties, notably under severe SA stress conditions, whereas the *OsNHX1* expression in both rice varieties increased by 5.5-fold (Pokkali) and 4.6-fold (PTT1) compared with that under the control conditions (Figure 4E). The expression level of *OsNHX1* in Pokkali shoots was lower than that in PTT1 roots under each treatment (Figure 4E). Simultaneously, the *OsSOS1* expression levels in Pokkali and PTT1 roots remained unchanged under SA stress conditions (Figure 4F). However, the *OsHKT2;1* expression in the roots was significantly downregulated in both rice varieties (Figure 4G). This finding implies that Na^+^ uptake via OsHKT2;1 in the roots was inactive under SA stress conditions.

### 2.4. Gene Expression Related to K^+^ Acquisition and Transport

**Figure 5:**
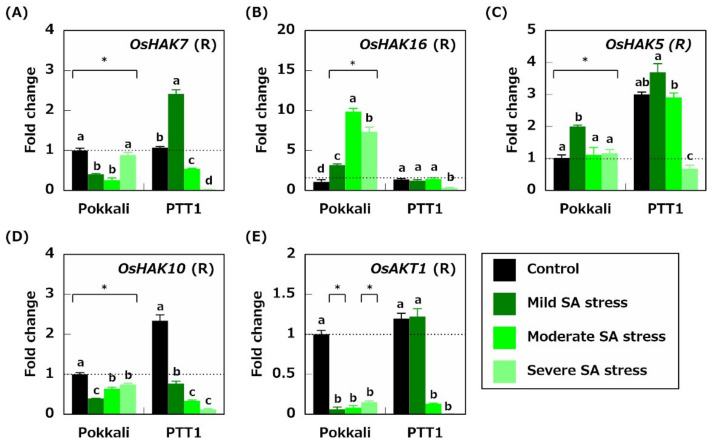
Relative expression of genes encoding K transporters *OsHAK7* (**A**), *OsHAK15* (**B**), *OsHAK5* (**C**), *OsHAK10* (**D**), and *OsAKT1* (**E**) in the roots of Pokkali and PTT1. The number of gene transcripts is evaluated based on that of Pokkali under control conditions. Similar letters indicate no significant differences between the treatments in each variety (*p* < 0.05). Asterisks show significant differences between Pokkali and PTT1 in each treatment (*p* < 0.05).

To explore the K^+^ absorption mechanisms in the roots under SA stress conditions, the expression levels of the genes encoding K^+^ channels and transporters (*OsAKT1, OsHAK5, OsHAK7, OsHAK10,* and *OsHAK16*) were analyzed. The *OsHAK7* expression in Pokkali roots under mild and moderate SA stress treatments was inhibited by 59.7% and 74.3%, respectively, compared with that under the control conditions (Figure 5A). In PTT1 roots, the *OsHAK7* expression was doubled under mild SA conditions; however, it was downregulated by 49.8% and 97.8% under moderate and severe SA stress conditions, respectively (Figure 5A). The *OsHAK16* expression levels in Pokkali roots were increased by 2.9-, 9.1-, and 6.8-fold under mild, moderate, and severe SA stress conditions, respectively, compared with those under the control conditions (Figure 5B). Meanwhile, the *OsHAK16* expression was not upregulated in PTT1 roots under mild and moderate SA stress conditions (Figure 5B). Alternatively, it was significantly inhibited by 80.0% under severe SA stress conditions than under the control conditions (Figure 5B). Furthermore, the *OsHAK5* expression levels in Pokkali roots under mild SA stress conditions were twice as high as those in Pokkali roots under the control conditions (Figure 5C). While, in PTT1, the *OsHAK5* expression increased by 2-fold under mild SA stress conditions; however, it decreased by 0.2-fold under severe SA treatments (Figure 5C). Similarly, the *OsHAK5* expression level in PTT1 roots under the control conditions was 3-fold higher than that in Pokkali roots under the control conditions (Figure 5C). However, the expression of *OsHAK10* and *OsAKT1* in Pokkali and PTT1 roots did not significantly increase under SA stress conditions (Figure 5D,E).

### 2.5. Genes Related to Fe Acquisition

**Figure 6:**
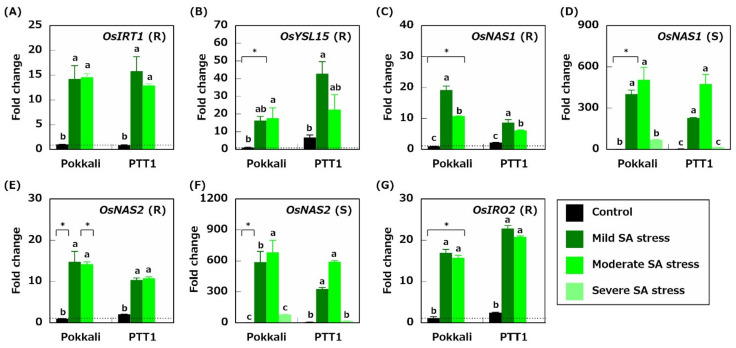
Relative expression of Fe acquisition-related genes; *OsIRT1* (Fe (II) ion transporter) in the roots (**A**), *OsYSL15* (Fe^3+^-DMA transporter) in the roots (**B**), *OsNAS1* (nicotianamine synthases 1) in the roots (**C**) and shoots (**D**), *NAS2* (nicotianamine synthases 2) in the roots (**E**) and shoots (**F**) and *OsIRO2* (transcription factor) in the roots (**G**) of Pokkali and PTT1. The number of gene transcripts is evaluated based on that of Pokkali under control conditions. The concentration of each element in the roots of both rice varieties under severe SA conditions is not determined because of divalent metals deposition on the root surface. Data are the means of 3 replicates ± S.E. Similar letters indicate no significant differences between the treatments in each variety (*p* < 0.05). Asterisks show significant differences between Pokkali and PTT1 in each treatment (*p* < 0.05).

The expression patterns of genes related to Fe acquisition, including *OsIRT1, OsYSL15, OsIRO2, OsNAS1,* and *OsNAS2*, were also determined. The expression levels of *OsIRT1*, encoding an Fe (II) ion transporter, was increased in the roots of Pokkali and PTT1 under mild (14.2- and 18.4-fold) and moderate (14.5- and 15.0-fold) SA stress conditions compared with those under the control conditions (Figure 6A). Similarly, *OsYSL15*, encoding an Fe^3+^-DMA transporter, YSL15, was highly expressed in the roots of Pokkali and PTT1 under mild and moderate SA stress conditions (Figure 6B). The *OsNAS1* and *OsNAS2* expressions encoding nicotianamine synthases 1 and 2 were also upregulated in the roots of both rice varieties under mild and moderate SA stress conditions (Figure 6C,E).

Furthermore, the *OsNAS1* expression levels in the roots of both rice varieties under moderate SA stress conditions were lower than those under mild SA stress conditions (Figure 6C). In Pokkali and PTT1 shoots, *OsNAS1* and *OsNAS2* were also highly expressed in the mild and moderate SA stress conditions (Figure 6D,F). However, the *OsNAS1* and *OsNAS2* expressions in the shoots of both rice varieties were repressed to the same levels as those in the control plants under severe SA stress conditions (Figure 6D,F). *OsIRO2,* a transcription factor regulating Fe(III)-DMA uptake and Fe translocation via *OsNAS1* and *OsYSL15*, was also highly expressed in the roots of Pokkali and PTT1 under SA stress conditions. Here, the expression levels of *OsIRO2* under mild SA stress conditions were the highest (14.8- and 9.3-fold) under all SA stress conditions (Figure 6G).

## 3. Discussion

SA stress is one of the main factors affecting rice production in several countries, such as Thailand. Nevertheless, only a few studies on SA tolerance in rice were conducted recently. In this study, Pokkali, a landrace tolerant to neutral salts, and PTT1, a cultivar sensitive to neutral salts, were used to determine how these varieties differ in their responses to SA stress in terms of nutrient absorption. Physiological analysis revealed that the growth of both varieties was significantly reduced under all SA stress conditions and that the magnitude of growth reduction of PTT1 was higher than that of Pokkali (Figure 1D). Furthermore, the growth of Pokkali and PTT1 shoots was most reduced under severe SA stress conditions than under mild and moderate SA stress conditions (Figure 1D). SA stress also increased the Na concentrations and decreased the K concentrations in both rice varieties (Figure 2); however, Pokkali maintained the lower Na/K ratio in the shoots than PTT1 (Table 1). The Fe concentrations in Pokkali and PTT1 roots were also decreased under SA conditions; however, the difference was not statistically significant (Figure 3C).

It has been established that salinity-tolerant varieties, such as Pokkali, activated salinity-tolerance mechanisms under salt-stress conditions. Under SA stress conditions, Pokkali maintained lower Na concentrations in the roots and shoots than PTT1, indicating that the mechanisms of Na exclusion from the xylem transpiration stream to xylem parenchyma cells in Pokkali roots were relatively active under SA stress conditions. It has been proposed that this activity is due to the restriction of Na accumulation in shoots caused by the Na^+^ transporter OsHKT1;5 in rice, which mainly localizes Na^+^ in xylem parenchyma cells of roots [8]. Therefore, the contribution of OsHKT1;5 to Na exclusion was evaluated by quantitative expression analysis. Figure 4A demonstrated that the expression of *OsHKT1;5* was suppressed in both varieties under SA stress. However, the *OsHKT1;5* expression in Pokkali roots was higher than that in PTT1 roots under the control and SA stress conditions, indicating that Pokkali can maintain a relatively higher *OsHKT1;5* activity than PTT1. This result is proposed to be one reason for the lower Na concentrations in Pokkali shoots than in PTT1 under SA stress conditions. In addition to the differences in *OsHKT1;5* expressions in the roots, the *OsHKT1;5* expression in Pokkali shoots under severe SA stress conditions was significantly increased, unlike in PTT1. These results also indicate that Pokkali exhibits higher SA tolerance than PTT1 under severe SA stress conditions. However, the Na concentrations in both rice varieties were significantly higher under severe SA stress conditions than under mild and moderate stress conditions (Figure 2A,B). This finding also reveals that high pH stress deactivates the Na exclusion mechanisms in both rice varieties. As a result, Na was considerably accumulated in whole plants. Chuamnakthong et al. demonstrated that rice accumulated more Na under high pH than under low pH SA stress conditions [31]. Therefore, the findings of this study indicate a common response of rice to SA stress. Alternatively, the *OsNHX1* expression was upregulated in the roots and shoots of both rice varieties under all SA conditions and was higher under severe SA conditions than under mild and moderate SA stress conditions (Figure 4D,E). Thus, the upregulation of the *OsNHX1* expression in the shoots corresponded to an increase in the Na concentrations in the shoots. Accordingly, it is possible to infer that rice enhances tissue tolerance to cope with the increase in Na in the shoots under SA stress conditions.

In contrast to the Na concentrations, the K concentrations in the roots and shoots of Pokkali and PTT1 decreased under all SA stress conditions and tended to be lower as stress worsened (Figure 2C,D), suggesting that high pH under SA stress reduces the K^+^ absorption ability of both rice varieties. The K concentrations in the roots and shoots of PTT1 were also significantly lower than those of Pokkali under SA stress conditions (Figure 2C,D). *OsHAK16* was upregulated in the roots under SA stress conditions in Pokkali but not in PTT1 (Figure 5B). *OsHAK16* encodes a K^+^ transporter located on the plasma membrane at the root epidermis; therefore, OsHAK16 participates in K^+^ absorption from the soil [33]. It has been reported that OsHAK16 is highly expressed in the roots under salinity conditions and contributes to salinity tolerance by maintaining a high rate of root K^+^ uptake [18]. Under SA stress conditions, OsHAK16 is likely to help maintain the K concentration in the roots of Pokkali. However, in PTT1, the expressions of genes encoding K^+^ channels and transporters (*OsAKT1, OsHAK7, OsHAK5*, and *OsHAK10*) were significantly downregulated under SA stress conditions, especially under severe SA stress conditions (Figure 5A,C–E). This finding suggests that OsAKT1, OsHAK7, OsHAK5, and OsHAK10 do not contribute to the maintenance of stable K^+^ absorption under SA stress conditions.

Fe is necessary to chlorophyll synthesis and photosynthesis in plants. Plants enhance their Fe acquisition capacity under low Fe conditions to cope with Fe deficiency [27]. Therefore, Fe-deficient rice enhances the Fe acquisition mechanisms by expressing the genes related to Fe uptake and translocation [25]. Under SA stress conditions, rice also activates the Fe acquisition mechanisms. In this study, the Fe concentrations in the roots of both rice varieties tended to decrease under SA stress conditions, especially in Pokkali (Figure 3C). The expressions of genes related to Fe uptake and translocation (*OsIRT1, OsYSL15, OsNAS1, OsNAS2,* and *OsIRO2*) were significantly upregulated in the roots of Pokkali and PTT1 under SA stress conditions (Figure 6A–C,E,G). However, the Fe concentrations in the shoots did not significantly decrease in Pokkali and PTT1 under SA stress conditions (Figure 3D). Furthermore, the *OsNAS1* and *OsNAS2* expressions in the shoots were significantly increased under mild and moderate SA stress conditions (Figure 6D,F). *OsNAS1* and *OsNAS2* encode nicotianamine synthases 1 (OsNAS1) and 2 (OsNAS2), which catalyze the conversion from *S*-adenosyl methionine to nicotianamine [34]. Furthermore, nicotianamine plays vital roles in the long-distance transport of Fe [35]. These findings and facts indicate that nicotianamine biosynthesis contributes to Fe homeostasis in rice under SA stress conditions. However, considering the maintenance of the Fe concentrations in the shoots and reducing the Fe concentrations in the roots under SA stress conditions (Figure 3C,D), it is proposed that Pokkali and PTT1 activate the Fe translocation mechanisms in the shoots to cope with Fe deficiency under SA stress conditions, as well as enhance Fe uptake in the roots.

The Ca, Mn, Zn, and Cu concentrations in Pokkali and PTT1 roots exposed to SA stress conditions dramatically increased (Figure 3A,G,I,K). Contrarily, the Ca, Mn, Zn, and Cu concentrations in Pokkali and PTT1 shoots significantly decreased under SA stress conditions (Figure 3B,H,J,L). It is speculated that Ca, Mn, Zn, and Cu in the roots of rice under SA conditions were not translocated to the shoots due to SA stress. Therefore, these concentrations increased in the roots, whereas those in the shoots decreased under SA stress conditions. However, further studies are needed to elucidate the mechanisms of Ca, Mn, Zn, and Cu translocation inhibition under SA stress conditions.

This study demonstrated that Pokkali, a salinity-tolerant landrace, is relatively SA-tolerant compared with PTT1, a salinity-sensitive rice variety. However, Pokkali’s SA tolerance was decreased under severe SA stress conditions, making it undesirable to be used as a parent to generate new SA-tolerant rice varieties. Therefore, to successfully cultivate rice in fields prone to SA stress, it is crucial to find new rice varieties that exhibit SA stress tolerance and to understand the mechanisms of SA stress tolerance.

## 4. Materials and Methods

### 4.1. Plant Materials and Methods

In this study, two rice varieties were used: Pokkali, a landrace tolerant to neutral salts, and PTT1, a cultivar sensitive to neutral salts. Cultivation was performed at the Faculty of Agriculture of Kasetsart University, Thailand. After surface sterilization in hot water at 60 °C for 10 min, the rice seeds were soaked in tap water at room temperature for 2 d for germination. The germinated seeds were then transplanted and cultivated for 10 d on nylon-mesh nets floating on a half-strength Kimura B hydroponic solution. This solution contains the following macronutrients: 0.18-mM (NH_4_)_2_SO_4,_ 0.27-mM MgSO_4_7H_2_O, 0.09-mM KNO_3_, 0.09-mM KH_2_PO_4_, and 0.18-mM Ca(NO_3_)_2_ 4H_2_O. The solution used also contained the following micronutrients: 19-µM FeSO_4_ 7H_2_O (used instead of Fe-EDTA), 48-µM H_3_BO_4_, 9-µM MnSO_4_ 5H_2_O, 0.3-µM CuSO_4_ 5H_2_O, 0.7-µM ZnSO_4_ 7H_2_O, and 0.09-µM Na_2_MoO_4_ 2H_2_O. Subsequently, 10-day-old rice plants were subjected to SA stress as follows: 50-mM Na + pH 7.0 (50-mM NaCl: mild SA stress), 50-mM Na + pH 8.0 (47-mM NaCl + 3-mM NaHCO_3_: moderate SA stress), and 50-mM Na + pH 9.0 (30-mM NaCl + 20-mM NaHCO_3_: severe SA stress). Moreover, 0-mM Na + pH 5.5 treatment was used as the control. The pH of the hydroponic solution, measured using a pH meter, was adjusted daily using 2 N HCl or 2 N NaOH. Then, the hydroponic solution was replaced every 5 d, and tap water was added daily to maintain the volume of the hydroponic solution.

### 4.2. Sampling

Sampling was conducted 7 d after the initiation of the SA stress treatments. The roots and shoots of rice plants were washed with tap water to eliminate salts derived from the hydroponic solution. The root and shoot length, root number, total root length, and SPAD (an indicator of chlorophyll content) were measured. Subsequently, the rice samples were separated into the roots and shoots and dried for 3 d at 70 °C. Dried samples were used to measure the dry weights. Furthermore, four replicates were used for each variety. Samples for gene expression analysis were stored at −60 °C after inactivation of RNase by soaking in the nucleic acid preservation buffer according to the method described by Camacho et al. [36].

### 4.3. Element Analysis

Essential element concentrations in the roots and shoots of rice were measured using an inductively coupled plasma optical emission spectrometer. Here, dried samples (100 mg) were powdered using a crusher and acid-digested in 2-mL of the digestion mixture (HNO_3_:HCl:HClO = 5:1:2) at 150 °C for 30 min and 200 °C for 5 h using a heat block.

### 4.4. Extraction of Total RNA, Reverse Transcription Reaction, qRT-PCR Using SYBR Green

Total RNA was extracted using RBC Total RNA Extraction Kit Mini (Plant) (RBC Bioscience, Birmingham, UK). Frozen samples (100-mg) were homogenized using a crusher. The homogenized samples were placed in a microtube and mixed with 500-µL RB buffer and 5-µL 2-mercaptoethanol and left to stand for 5 min at room temperature after vigorous mixing for 30 s. The samples were then centrifuged at 10,000× *g* for 2 min at 23 °C. Subsequently, the supernatant was added to a filter column and centrifuged at 10,000× *g* for 2 min at 23 °C. Then, the filtrate was added to 250-µL of 100% (*v/v*) ethanol, after which the mixture was transferred to an RB column. The RB column was centrifuged at 10,000× *g* for 1 min at 23 °C. Furthermore, R-W1 buffer (200-µL) was added to the RB column, and the column was centrifuged again at 10,000× *g* for 1 min. After the addition of 100-µL DNaseI solution to the column, genomic DNA was digested for 20 min at room temperature. The R-W1 buffer was then added again, after which the RB column was centrifuged at 10,000× *g* for another 1 min at 23 °C. Then, the column was drained via centrifugation at 10,000× *g* for 3 min at 23 °C, and total RNA was eluted by adding 20-µL RNase-free water. The concentration of total RNA was measured using a NanoDrop One spectrophotometer. Subsequently, total RNA (1 µg) in a PCR tube was incubated for 5 min at 65 °C and cooled on ice for 2 min. Then, 5× Master Mix was added to the PCR tube. Reverse transcription was then performed using a thermal cycler as follows: incubation at 37 °C for 15 min, 50 °C for 5 min, and 98 °C for 5 min.

qRT–PCR was then performed to analyze the expression of genes related to the Na^+^ and K^+^ transport, including Fe acquisition. Here, *OsUBC* and *Os25SrRNA* were used as reference genes in the roots and shoots, respectively. qRT–PCR was then conducted using THUNDERBIRD SYBR qPCR Mix (Toyobo Co., Ltd., Osaka, Japan) and an ABI Step One Real-Time PCR System (Applied Biosystems, Foster City, CA, USA). The reaction mixture consisted of 7.5-µL SYBR Mix, 0.3-µL 50X ROX reference dye, 1.5-µL forward primer, 1.5-µL reverse primer, 1-µL cDNA, and 3.2-µL RNase-free water. Subsequently, qRT–PCR was performed as follows: initial incubation at 95 °C for 1 min, 40 cycles of denaturation at 95 °C for 15 s, and extension at 60 °C for 60 s, followed by melting curve analysis. The relative expression levels of gene transcription were calculated using the comparative 2^−ΔΔCT^ method. Data were the average of three replicates.

### 4.5. Statistical Analysis

Two-way ANOVA and the Tukey–Kramer test were employed to detect significant differences between treatments using R software. F-test and Student’s *t*-test, as well as Microsoft Excel were used to compare significant differences between the two rice varieties. Statistical significance was set at *p* < 0.05 for all variables.

## 5. Conclusions

In conclusion, this study suggested that Pokkali, a salinity-tolerant landrace, was relatively SA-tolerant compared with PTT1, a salinity-sensitive variety, owing to its ability to restrict Na^+^ accumulation in the shoots. The maintenance of a lower Na/K ratio in the shoots is proposed to be achieved by higher expressions of *OsHKT1;5*, *OsNHX1*, and *OsHAK16* in the roots of Pokkali under SA conditions. Both rice varieties also enhanced the expression of Fe deficiency-responsive genes and increased Ca, Mn, Zn, and Cu accumulation in the roots but decreased the concentrations of these minerals in the shoots. Thus, high rhizospheric pH influenced nutrient uptake and translocation from the roots to the shoots in rice.

## Figures and Tables

**Table 1:** Effects of saline-alkaline stress (mild, moderate, and severe) on the Na/K ratio of the roots and shoots of Pokkali and PTT1. Data are the means of 4 replicates ± S.E. Similar letters indicate no significant differences between the treatments in each variety (*p* < 0.05). Asterisks show significant differences between Pokkali and PTT1 in each treatment (*p* < 0.05).

Treatment	Cultivar	Root	Shoot
Control	Pokkali PTT1	0.56 ± 0.02 ^c^ * 0.34 ± 0.01 ^c^	0.05 ± 0.01 ^c^ 0.06 ± 0.00 ^c^
Mild SA stress (50 mM Na + pH 7.0)	Pokkali PTT1	5.76 ± 0.17 ^b^ 5.80 ± 0.71 ^b^	2.16 ± 0.08 ^b^ * 3.76 ± 0.48 ^b^
Moderate SA stress (50 mM Na + pH 8.0)	Pokkali PTT1	7.14 ± 0.15 ^b^ 6.87 ± 0.34 ^b^	2.53 ± 0.14 ^b^ * 4.66 ± 0.19 ^ab^
Sever SA stress (50 mM Na + pH 9.0)	Pokkali PTT1	11.80 ± 0.89 ^a^ * 12.85 ± 0.72 ^a^	3.55 ± 0.24 ^a^ * 6.19 ± 0.64 ^a^

## Data Availability

All data, tables and figures in this manuscript are original.
